# Mechanism of Nervous Action

**Published:** 1857-11

**Authors:** J. Adams Allen

**Affiliations:** Formerly Pofessor of Therap., Mat. Med. and Juris. in the Indiana Medical College; and late Professor of Physiology and Pathology, and acting Prof. of Mat. Med. in the University of Michigan


*Mechanism of Nervous Action.* By J. Adams Allen, A. M., M. D., Formerly Pofessor of Therap., Mat. Med. and Juris. in the Indiana Medical College; and late Professor of Physiology and Pathology, and acting Prof. of Mat. Med. in the University of Michigan.

The readers of the London Lancet will recollect that W. Tyler Smith, in the year 1848, pointed out the remarkable synergic relations of the three great organs of the sexual system,—uterus, mammæ and ovaria. Had he not been blinded by the term "excito-motory," it seems impossible to believe but that he would then have discovered the real mechanism of the "synergies." (Vide Lord. Lan., Gct. 1848, p. 327.)

One point only I here notice:—Tyler Smith urges in that series of lectures, that the motions attributed to the *fœtus in utero*, were, in fact, caused by motion of the uterus itself. There is no nervous connection between the mother and child—hence he argued the excito-motor influence, well known to give rise to these movements, must act upon the muscular tissues of the uterus. But careful investigation convinced me that the fœtus was itself the moving object.

The corollary is clear—the nervous are induced a change in the blood which supplied the placenta, and this modified blood caused muscular contraction in the fœtus. The *modus*, the same as though moving the muscles of the mother.

Why does parturition occur at a fixed period?

Because the changes in the blood of the mother occasioned by the full developement of the fœtus produce that particular molecular change in the uterine muscle which necessitates contraction. The molecular change is the cause.

The same blood-modification may occur from accidental causes, or through the medium of the nervous arc.

Why does application of the child to the breast provoke the mammary secretion? Is it necessary to introduce the motor before the secretory effect? Is it philosophical? Not at all.

Constant molecular changes, the necessary condition of life, keep up what is called "passive contraction," or the *tone* of muscles, but the exigencies of animal life require something more beyond this; something which will *intensify* these changes to suit the wants of the animal. The nervous system meets this demand, but not by the introduction of new modes of operation in the moving tissues. The molecular changes are varied by the influence of the nervous system.

The various functions of vegetable life are carried on under nearly identical conditions: the organs, therefore, have no need of internuncii. But when animal life comes in to vary the conditions, then internuncii become necessary. But nature chooses uniformity, and thus no new machinery or unparalleled aparatus is made use of. Synergic organs reciprocally respond to the simple nervous arc.

Much confusion has arisen among physiologists by vague attempts to localize a particular nervous centre. The true centre is the nerve-vesicle, and may radiate its influence to yet another point, which again becomes a centre. The nerve fibre creates nothing. Collectively the fibres serve the purpose of bringing all the vesicles together, and yet keeping them apart.

It is proved that nervous impulses will pass by vesicles in contact as well as by nerves of commissure. The white fibre insulates and localizes the effect—the gray more nearly analogous to the vesicle, diffuses it almost indefinitely. This is shown by their several connections and by observation.

The points of contact of the so-called "sympathetic," and the cerebro-spinal systems are limited, yet they do exist; and gray and white fibres may intermingle, as we see fibrillæ of striated and non-striated muscular tissue side by side.

The ganglionic chain is little connected with the cerebro-spinal axis, simply because it is unnecessary, and nature never does a needless work. But this shows no real diversity of operation. As well might we exclaim against the optic nerve and vesicles because they are not intimately connected with a lumbar spinal nerve and its fused ganglion.

The outward expansion of the nerves of special sense, it is now believed, consists of vesicular neurine. It is quite probable that this is the general fact for all recipient nerves. The pecular structure of this vesicular expansion fits it for undergoing changes on the application of its particular stimulous, and that only. Transmitted inward by the conducting tissue, it developes there another change, which may be reflected to several different points, according to the connections of the central vesicles--the central vesicles, in the latter instance, having the same relation to the second vesicular centre reached, that the outward vesicular expansion had to the first centre.

To take the optic nerve, for example, and trace the series of operations which may result from its action:

1. Retinal change; 2. central vesicular change at the "origin" of the optic nerve; 3. a "Reflex" upon the iris; b. upon the lachrymal gland; c. upon the blood vessels of the eye; d. Through the gray vesicles of the central nervous ganglia (fused) of the base of the brain, and by commissural fibres to gray matter of the convolutions, etc, of the cerebrum; e. ditto. to the cerebellar vesicles; f. through various nerves, vesicles and ganglia to muscular organs; g. to secreting organs.*

From careful study upon this topic, I am inclined to the belief that the gray vesicles upon the convulsions and anfractuosities of the brain have a similar relation to the commissural fibres and central nervous vesicles (constituting the true centres of volition, sensation, etc.) that the expansions upon the optic, and other nerves of special sense, do to their commissural fibres and the same centre.

The retinal cells respond only to their own stimulous; and their lesion, or that of their fibres, or central vesicles, gives rise to the subjective phenomena of vision.

In like manner the superficial cerebral vesicles respond only to their peculiar stimulous, and lesion of them or their commissural fibres of connection, etc., gives rise to the subjective phenomena of mind.

If there is anything of mind beyond the registered impressions of sense, it finds the organs of communication with the body through these vesicles, as light comes in by the retina, and sounds by the cells of the distal auditory expansion!

The force of mind once producing the vesicular change, the mechanism of its action thereafter is altogether analogous to that of physical stimuli!

In all are the commencing vesicle, the conducting fibre, and the recipient vesicle, which latter becomes the commencing vesicle of a new chain!

A correct idea of the *reflex phenomena* of mental nervous action, will explain, at once, about all of the real phenomena of "spiritualism," table-tipping, mesmerism, somnambulism, dreaming, etc.

Abstract metaphysics applied to psychology causes the whole thing to

---

* It will be noticed that in deference to common usage, I employ different terms for muscular and secreting organs, but it should be recollected that physiologically and histologically, they are homologous.

evaporate in subtleties; direct physiological investigation brings the whole subject to the simplest possible concrete.

I have said elsewhere that the "new principle," or "doctrine," is but a corollary from what was previously known. This is true of all discoveries by the inductive method.

It was a known fact that diseases or disturbances of the nervous centres, deranged both motion and secretion in the parts supplied from those centres.

The term functional disease was either meaningless, or it involved in many instances reflex phenomena.

The terms *centric* and *ex-centric* causes of internal disorder could find no other explanation.

The entire doctrine of "revulsion" was vague and indefinite without this transparent statement.

The structure of muscular tissue, its cell formation and relation to nervous tissue demanded this view.

The doctrine of "sympathies" was otherwise incapable. The discoveries in cytogenesis and histology, showing the analogy of development, nutrition, and decay of muscular and glandular organs pointed definitely to this idea.

Even though the opinion of M. Hall that muscular irritability is dependent upon the cerebro-spinal system be still entertained, there is no escape from the conclusion that the blood and fluids must be the medium of action.

In October, 1849, in the case of a gentleman on trial for homicide of his son, I gave evidence upon the question of insanity, and argued the presence of diabetes as indicative of disease of the nervous centre. The other indications, and the event, confirmed the diagnosis. (Vide Bost. Med. and Surg. Jour., Vol. 42, p 441.) Few writers, indeed, have been found hardy enough to deny the influence of disease of the nervous centres upon secretion. The corollary is obvious, that nerves which induce changes in these nervous centres will, secondarily, induce changes in parts receiving nervous supply from those centres.

Irritate the ulnar nerve at the elbow, and sensation is referred to the ring and little finger;—continue the irritation and actual inflammation will ensue in the parts experiencing sensation. Where is the line of demarkation between the sensory and secretory here? There is precisely the same effect in case of reflected sensation elsewhere.

Probably the largest number of cases of neuralgia are examples of what might be called excito-sensitory action.

Is there philosophical propriety in inventing the term ?

The central truth is that the nerve vesicle undergoing change, induces changes in structures with which it is in connection, by conducting nerve fibres, or vesicles—the expression or form of that change depends on the structure reached.

The nerve vesicle may be primarily undergoing change, or by the m e dium of an incident fibre.

---

*Letter from W. R. Marsh, A. M., M. D.,* Professor of Chemistry Materia Medeica, and Toxicology, Medical Department of Iowa University.

Keokuk, Iowa, August 13, 1857.

My Dear Doctor :—

Sickness at home, and the urgent pressure of other business must be my apology for delay in replying to you.

To your interrogations I reply but briefly because opportunity is wanting for speaking more at length now, although should you wish it, I will write more fully at another time.

*First.* The manuscript lectures to which you refer, and from which the transcript mentioned is taken, were written by you during the summer of 1848, and publicly delivered before the class of the Indiana Medical College during the session of 1848-9.

I am possitive as to the date of the writing and delivery, and the identity of the manuscript, because I was then reading medicine under your instruction; and, as we were not merely teacher and pupil, but intimate friends, it was permitted me to peruse your lectures from day to day as you progressed with their preparation. During the session of 1848-9 I occupied the same private room with yourself, and it was my custom each evening to look over the lecture you had given during the day, for the purpose of fixing the subject more fully in the memory, and also of correcting, if need be, my notes of the same. Hence I could not but remember, and if I failed to do so, the notes thus taken would be sufficient to revive remembrance.

*Second.* As to what you taught, I reply: That in addition to what you had written in explanation of your views of the *modus operandi* of escharotics and revulsives generally, you were wont to enlarge extemporaneously, appealing to analogy and known facts to sustain your position.

I may sum up your remarks, in brief, as follows :

*Secretion by glandular or secernent organs* is, in relation to the reflex nervous system the analogue of *motion* in muscular tissue. Both are operations of double nervous arcs. Impressions upon efferent nerves, conveyed to nerve centres, produce changes therein, thence the influence is conveyed through efferent fibres to the organ or organs in direct nervous connection (through the double arc) with the part where the first impression is made, and at the distal extremities of these efferent nerves, molecular changes are produced. These changes result in the production of motion, sensation, or secretion, just as the organ has for its proper function the one or the other. "Contiguous sympathy" may be thus explained, as also the "sympathies" of organs topographically or anatomically remote. And I remember you adduced as an argument in support of these views, the self-same instances advanced by Dr. Campbell—the "*sympathies*" displayed in dentition; as also the influence of impressions made upon other parts, as the feet, mammæ, etc., upon the menstrual secretion Here, both the motor and secretory influence were apparent accordingly as the character of the uterus assumed more or less of the office of a muscle or of a secretory organ.

The above illustrations, made by you, of your views, I give; and although imperfectly as representing the manner of your treating the subject, yet I hope they will be sufficient for your present purpose. The utmost brevity is all my time will now permit.

*Third.* You taught the same in the winter of 1849-50, but more fully than in the preceding course As to your after teachings, I know nothing positively except as follows:

In the summer of 1851 or '52 you made me a visit, and while sitting in my office with me, the conversation fell upon the comparative pressure in lecturing upon Mat. Med or Physiology. I asked you how it fared with your two *enthusiasms*, viz: your views of the office of the reflex system of the nerves, and the catalytic action of medicines, when you replied that you had a better opportunity in lecturing upon both branches to develop the former fully to the minds of the class, for that when upon the "excito-motor" nerves, it was easy to show how motion and secretion were analogous results of the same causes. You expressed yourself as more than ever confident of the justice of your views, and expressed some astonishment that writers upon therapeutics should have failed to notice a matter so important in a pathological sense, and as an index of therapeutic applications.

Finally, your teachings have so far influenced *my mind*, at least, as to induce me to teach the same to our classes here in my lectures upon therapeutics. To the fact that I was under your instruction from the first of my medical study, may doubtless be attributed my not noticing the fact that it was something new in physiology, although I was aware that the application of the idea, as made by you, was new, and I have so remarked in my teachings, giving you the credit.

Hoping that you may be able to prove to the satisfaction of the profession, what I have no doubt you are entitled to, the credit of being the first to arrive at and promulgate "*the idea*" although not the "designation" of the "excito-secretory" system of nerves; and that you may be ever successful in your efforts for the good of medical science, I am,

Sincerely yours, WELLS R. MARSH.

Professor J. ADAMS ALLEN, M. D., Kalamazoo, Mich.

*(To be Continued.)*

